# Partitioning of functional gene expression data using principal points

**DOI:** 10.1186/s12859-017-1860-0

**Published:** 2017-10-12

**Authors:** Jaehee Kim, Haseong Kim

**Affiliations:** 1 Department of Statistics, Duksung Women’s University, Seoul, South Korea; 2 Korea Research Institute of Bioscience and Biotechnology (KRIBB), Daejeon, South Korea

**Keywords:** Fourier coefficients, Legendre polynomials, *Escherichia coli* Microarray expression data, K-means clustering, Principal points, Silhouette, Yeast cell-cycle data

## Abstract

**Background:**

DNA microarrays offer motivation and hope for the simultaneous study of variations in multiple genes. Gene expression is a temporal process that allows variations in expression levels with a characterized gene function over a period of time. Temporal gene expression curves can be treated as functional data since they are considered as independent realizations of a stochastic process. This process requires appropriate models to identify patterns of gene functions. The partitioning of the functional data can find homogeneous subgroups of entities for the massive genes within the inherent biological networks. Therefor it can be a useful technique for the analysis of time-course gene expression data. We propose a new self-consistent partitioning method of functional coefficients for individual expression profiles based on the orthonormal basis system.

**Results:**

A principal points based functional partitioning method is proposed for time-course gene expression data. The method explores the relationship between genes using Legendre coefficients as principal points to extract the features of gene functions. Our proposed method provides high connectivity in connectedness after clustering for simulated data and finds a significant subsets of genes with the increased connectivity. Our approach has comparative advantages that fewer coefficients are used from the functional data and self-consistency of principal points for partitioning. As real data applications, we are able to find partitioned genes through the gene expressions found in budding yeast data and *Escherichia coli* data.

**Conclusions:**

The proposed method benefitted from the use of principal points, dimension reduction, and choice of orthogonal basis system as well as provides appropriately connected genes in the resulting subsets. We illustrate our method by applying with each set of cell-cycle-regulated time-course yeast genes and *E. coli* genes. The proposed method is able to identify highly connected genes and to explore the complex dynamics of biological systems in functional genomics.

## Background

Discovering which genes are functioning and how they express their changes at each time is a necessary and challenging problem in understanding cell functioning [10]. The large number of genes in biological networks makes it complicated to analyze to understand their dynamics. The mathematical and statistical modelling of these dynamics, based on the gene expression data, has become an intensive and creative research area in bioinformatics.

Statistical models can find genes with similar expression profiles whose functions might be related through statistics or biology. Our approach has the assumption that specific curve form exists for each gene’s trajectory and for each partition of these gene curves.

The observations of gene expressions are curves measured according to time on each gene. We can then call the observed lines of genes functional data because an observed intensity is recorded at each time point on a line segment. Functional data analysis is possibly considered a suitable method to model these gene curves [53].

Clustering algorithms are utilized to find homogeneous subgroups of gene data with both supervised or unsupervised [1]. For functional data, clustering algorithms based on the functional structure are also useful to find representative curves in each partition.

To obtain more knowledge about biological pathways and functions, classifying genes into characterized functional groups is a first step. Many methods of analysis, such as hierarchical clustering [34], K-means clustering [48, 52], correlation analysis [22, 24] and support vector machines (SVM) [6] classification, can be used to classify temporal gene profiles. Model-based clustering with finite mixture [29] was done based on probabilistic models [4, 13, 20, 28, 54]. Recently time-course gene expression data is often clustered in the relation between successive time points [7, 51, 55]. Yeast gene network is investigated for possible functional relations [31]. Fourier transformation is also incorporated in clustering and compared with Gaussian process regression (GPR) [21]. We use the word partitioning instead of clustering since we use a principal points partitioning technique. After partitioning, the subsets are often but not always normally disjoint.

In this paper, we use Legendre orthogonal polynomial system and principal points to obtain functional partitions. Analysis can be accomplished through extracting representative coefficients via data dimension reduction and finding principal points. Connectedness and silhouette values are computed for partition validity measure. An efficient way to deal with such gene data is to incorporate the functional data structure and to use a partitioning technique.

As a smooth stochastic functional process, the observed gene expression profiles have the covariance function which can be expressed with smooth orthogonal eigen-functions based on functional principal components. The random part of Karhunen-Loeve representation of the observed sample paths serves as a statistical approximation of the random process.

Abraham et al. [1] proposed a partitioning procedure of functional data by B-splines. Kurata and Tang [23] investigated the properties of 2-principal points with the data from spherically symmetric distributions. Tarpey et al. [44] compared a growth mixture modeling and optimal partitioning with the principal points for longitudinal clinical trial data. Their simulation results indicated that the optimal partitioning worked better than the mixture model in a squared error, even if there is a covariate. Tarpey et al. [41] used the self-consistent partitioning with the functional data.

The *k*-principal points are defined as a set of *k*-points that minimizes the sum of expected squared distances from every point to the nearest point of the set. These *k*-principal points are mathematically equivalent to centers of gravity obtained by K-means clustering. Tarpey [42, 43] also extended and applied the principal points idea for functional data analysis (FDA).

In this paper, we handle the relation between clustering functional data and partitioning functional principal points. We propose to use self-consistent partitioning techniques for gene grouping based on curvature profiles as FDA. Some advantages in the use of FDA techniques for partitioning are:

(i) Tarpey [41] showed that partitioning random functions can be replaced by partitioning the coefficients of the orthonormal basis functions in finite Euclidean space if its approximation can be done based on a finite number of orthonormal basis functions. The orthonormal polynomials are estimated and partitioned ([39, 42–44]). Tarpey [41] proved that principal points of a Gaussian random function can be found in a finite dimensional subspace spanned by eigen-functions of the covariance kernel associated with the distribution.
(ii) For functional data, clustering algorithms are useful to find representative curves under the different modes of variation. Representative curves from a data set that can be found using principal points from a large collection of functional data curves [11, 37].
(iii) Principal points are special cases of self-consistent points. A set of *k*-points are self-consistent for a distribution if each of the points is the conditional mean of the distribution over its respective Voronoi region. K-means algorithm converges to a set of *k* self-consistent points of the empirical distribution if a set of *k*-points are self-consistent.

Partitioning based on interactions of genes is studied for the structure of genetic networks. In addition, statistical test and association rule approach represents another new strategy. Recently a statistical biclustering technique was proposed with applying on microarray data (gene expression as well as methylation) [25–27]. Consensus clustering is proposed via checking inter-method of clustering [40]. Recursive partition is also worked with classification trees to improve the precision of classification [56, 57]. To find the combinatorial marker [2, 3] integrated multiple data sources are surveyed in a comparative study. For yeast data a functional network partitioning was done [8].

Numerous research results on clustering microarray data which are mostly grouping common expression patterns. There are a few cases for partitioning genes with time-course regarded as functional data. In this research, we propose a new method for self-consistent partitioning of genes with functional gene expression data. The proposed method consists of two main steps. The first step is to represent each gene profile by functional polynomial representation. The second is to find principal points and appropriate partitions. We applied our method to simulated data and analyzed yeast gene microarray data and *Escherichia coli* data that resulted in partitioning with interpretable genes.

## Methods

### Model

Consider the gene expression data curve *Y*_*i*_(*t*) as a stochastic process at time *t*. Let *f*_*i*_(*t*) denote the expected expression at time *t* for the *i*th subject. The model with the functional data representation is

1 $$ {Y}_i(t)={f}_i(t)+{\varepsilon}_i(t),\kern1.25em i=1,2,\cdots, n $$

with

$$ {f}_i(t)={\beta}_{i0}{\overset{\sim }{\xi}}_0(t)+{\beta}_{i1}{\overset{\sim }{\xi}}_1(t)+{\beta}_{i2}{\overset{\sim }{\xi}}_2(t)+{\beta}_{i3}{\overset{\sim }{\xi}}_3(t)+{\beta}_{i4}{\overset{\sim }{\xi}}_4(t) $$

where each ${\overset{\sim }{\xi}}_j(t)$ corresponds to the normalized *ξ*_*j*_(*t*). For example, Legendre polynomials, as an orthonormal polynomial system, are expressed using Rodrigues’ formula as

$$ {\xi}_j(t)=\frac{1}{2^jj!}\frac{d^j}{dt^j}{\left({t}^2-1\right)}^j. $$

The first few Legendre polynomials are

$$ {\overset{\sim }{\xi}}_0(t)=1,\kern1.25em {\overset{\sim }{\xi}}_1(t)=t,\kern0.5em {\overset{\sim }{\xi}}_2(t)=\frac{1}{2}\left(3{t}^2-1\right), $$

$$ {\overset{\sim }{\ \xi}}_3(t)=\frac{1}{2}\left(5{t}^3-3t\right),\kern0.5em {\overset{\sim }{\xi}}_4(t)=\frac{1}{8}\left(35{t}^4-30{t}^2+3\right), $$

$$ {\overset{\sim }{\xi}}_5(t)=\frac{1}{8}\left(63{t}^5-70{t}^3+15t\right),\kern0.5em {\overset{\sim }{\xi}}_6(t)=\frac{1}{16}\left(231{t}^6-315{t}^5+105{t}^2-5\right), $$

and *ε*_*i*_(*t*) is an error function with mean 0, independent of each other term in the model. For each gene *β*_*i*0_, *β*_*i*1_, *β*_*i*2_, *β*_*i*3_, *β*_*i*4_ are regression coefficients based on Legendre polynomials. In the microarray experiment *Y*_*i*_(*t*) is the log gene expression of gene *i* at time *t*.

The curves given by the orthogonal polynomials are characterized by five coefficients, four of which are used to classify subjects. First, the coefficient *β*_1_ in (1) gives the overall trend in the outcome profile, then the derivative *f*_*i*_^′^(*t*) gives the rate of change in the expected outcome at time *t*. Parameter *β*_2_ is the coefficient of the quadratic polynomial providing a measure of concavity of the outcome curve. Parameter *β*_3_ as the coefficient of the cubic polynomial is a measure of curvilinearity and *β*_4_ as the coefficient of the quartic polynomial gives a measure of concavity of the outcome curve. The estimated polynomial coefficients have information about the underlying functional patterns and enable the automatic estimation of pattern functions.

## Partitioning functional gene curves

### Self-consistent partitions

Principal points and self-consistent points can be used for partitioning a homogeneous distribution. Principal points can be defined as a subset means for theoretical distributions.

For a set W = {***y***_1_, ***y***_2_, ⋯, ***y***_*k*_} the *k* distinct non-random functions in a function space L^2^, define

$$ {D}_j=\left\{\mathbf{y}\in {L}^2:||{\mathbf{y}}_j-\mathbf{y}|{}^2<||{\mathbf{y}}_i-{\mathbf{y}}^2|i\ne j\right\} $$

as a domain of attraction *D*_*j*_ of ***y***_*j*_ that consists of all **y** ∈ *R* ^*p*^. The sets of *D*_*j*_ are often referred to the Voronoi neighborhoods of ***y***_*j*_. The domains of attraction induce a partition as *D*_*j*_ via the pre-images *B*_*j*_ such as ∪*B*_*j*_ = *R* ^*p*^ where the boundaries have a probability of zero.

The set of optimal *k*-points is expressed in terms of mean squared error (MSE). A set of *k* points *ξ*_1_, *ξ*_2_, ⋯, *ξ*_*k*_ are principal points [8] for a random vector ***X*** ∈ *R* ^*p*^ if

$$ E\left(\underset{j=1,\cdots, k}{\min }||\mathbf{X}-{\upxi_j}^2\right)\le E\left(\underset{j=1,\cdots, k}{\min }|{\left|\mathbf{X}-{\mathbf{y}}_j\right|}^2\right) $$

for every set of *k* points ***y***_1_, ***y***_2_, ⋯, ***y***_*k*_. The optimal one-point representation of a distribution is the mean, which is corresponding to *k* = 1 principal point. For *k* > 1 principal points are a generalization of the mean from one to several points optimally representing the distribution. A nonparametric estimate for the principal points is obtained via K-means algorithm. Thus the *k*-points are mathematically equivalent to centers of gravity by K-means clustering.

The concept of principal points can be extended to functional data clustering. Tarpey [41–43] proved that principal points of a Gaussian random function can be found in a finite dimensional subspace spanned by eigen-functions of a covariance kernel associated with the distribution.

We derive functional principal points of orthonormal polynomial random functions based on the transformation.

A set {***ξ***_1_, ***ξ***_2_, ⋯, ***ξ***_*k*_} is self-consistent for a random vector ***X*** if

$$ E\left(\ \boldsymbol{X}\ \right|\boldsymbol{X}\in {D}_j\Big)={\boldsymbol{\xi}}_j,\kern0.5em j=1,\cdots, k. $$

A set of *k*-points is self-consistent if each of the points is a conditional mean in the respective domain of attraction. Principal points are self-consistent [8], but the converse is not necessarily true. Tarpey and Kinateder [46, 47] proved that self- consistent points of elliptical distributions exist only in a principal component subspace. Tarpey [41] proved the principal subspace theorem as follows. Suppose **X** is *p*-variate elliptical with *E*(***X***) = 0 and *Cov*(***X***) = **Σ**, then *v*, the subspace spanned by a self-consistent set of points is spanned by an eigenvector set of **Σ**. Principal points find the optimal partitions of theoretical distributions. It would be interesting to study principal points of theoretical distributions such as finite mixtures, for which cluster analysis is meant to work.

Tarpey [41] showed that principal points form symmetric patterns for the multivariate normal and other symmetric multivariate distributions. For symmetric, multivariate distributions several different sets of self-consistent points may exist and the optimal symmetric pattern of self-consistent points depends on the underlying covariance structure.

Cluster analysis is related to finding homogeneous subgroups in a mixture of distributions, it would be appropriate to give optimal cluster means to the principal points inspired by [24]. Cluster analysis methods are considered as purely data-oriented without a statistical model in the background in order to pragmatically find optimal partitions of observed data. It would be intriguing to further study principal points of theoretical distributions that reflect group structure, such as finite mixtures, due to their ability to find optimal partitions of theoretical distributions. Principal points may be used to define the best *k*-point approximations to continuous distributions.

Estimators of the principal points [11] can be obtained as cluster means form the K-means algorithm. Tarpey and Kinateder [46] examined the K-means algorithm for functional data and provided results on principal points for random functions. They proved that principal points of a Gaussian random function can be found in a finite dimensional subspace spanned by the eigen-functions of covariance kernel associated with distributions that can be extended to non-Gaussian random functions.

The self-consistent curves inspired by Hastie and Stuetzle [15] can be generalized to provide a unified framework for principal components, principal curves and principal points. A principal component analysis is proposed to identify important modes of variation among curves [17] with principal component scores demonstrating the form and extending variations.

Clustering algorithms are often used to find homogenous subgroups of entities depicted in a set of data. For functional data, clustering algorithms are also useful to find representative curves that correspond to different models of variation. Early work on the problem of identifying representative curves from a data set can be found based on the principal points [12, 17]. The concept of principal points to functional principal point was extended; subsequently, functional principal points of polynomial random functions were derived using orthonormal basis transformation [36].

Suppose {*f*_1_, *f*_2_, ⋯, *f*_*n*_} is a random sample of polynomial functions of the form (1) where the coefficient vector *β* = (*β*_0_, *β*_1_, *β*_2_, *β*_3_, *β*_4_)^′^ follows 5-variate normal distribution. The *L*_4_ version of the K-means algorithm can be run on the functions *f*_*i*_,  *i* = 1, ⋯, *n* to estimate principal points. The center of K-means clustering for the estimated coefficient vectors is based on the orthonormal transformation that constitutes the functional principal point; therefore, we consider K-means clustering for the Legendre polynomial coefficient vectors and for the Fourier coefficient vectors after Fourier transformation.

The K-means algorithm [47] provides that the Gaussian-based estimates coincide theoretically and the subspace containing a set of principal points must be spanned by the eigen-functions of the covariance matrix. Clustering functional data using an *L*_2_ metric on function space can be done by clustering regression coefficients linearly transformed based on the orthogonal system [45]. Clustering after transformation and nonparametric smoothing is suggested [36] without assuming independence between curves.

Estimated coefficient vectors can be used to obtain the principal points for partitioning. The subspace can be spanned by eigen-functions of the covariance kernel *C*(*s*, *t*) for β because the estimated coefficient vector can be a Gaussian random function. Eigenvalues and eigenvectors are then obtained from the covariance matrix of the estimated coefficients.

### Finding the number of partitions

One difficult problem in clustering analysis is to identify the appropriate number of groups for the dataset. As a nonparametric way [39] for choosing the number of clusters is based on distortion that measures the average between each observation and its closed cluster center. The minimum achievable distortion associated with fitting *K* centers to the data is

$$ \kern1.5em {d}_K=\frac{1}{p}\underset{{\boldsymbol{C}}_{1,\cdots, }{\boldsymbol{C}}_{\boldsymbol{K}\kern0.5em }}{\mathit{\min}}E\left[{\left(\boldsymbol{x}-{C}_{\boldsymbol{x}}\right)}^{\hbox{'}}\ {\varGamma}^{-1}\left(\boldsymbol{x}-{C}_{\boldsymbol{x}}\right)\right] $$

where Γ is the covariance matrix. If Γ is the identity matrix, distortion is a mean squared error.

The sample Legendre coefficients and the sample Fourier coefficients approximately follow the multivariate normal distribution; therefore, Gaussian mixture model-based clustering can be considered in addition to the number of partitions that can be chosen as a maximizer of the Bayesian Information Criterion (BIC).

### Choice of Legendre coefficients

xTo determine the value of *J*, the number of polynomials, we can consider several *J* values and BIC, assuming that each partition covariance has the same elliptical volume and shape. We surmise that a true optimal *J* value for all the genes may not exist because the known optimal *J* values are various for each gene function. Our experiments consider the feasible numbers of partitions and *J* values for their optimality with the corresponding dataset.

### Partition validation

The determination of the number of subsets (clusters) is an intriguing problem in unsupervised classification. To assess the resulting cluster quality various cluster validity indices are used. We consider silhouette measure proposed by [32] and connectivity in [14].

The silhouette width for the *i*th sample in the *j*th cluster is defined as:

$$ s(i)=\frac{b(i)-a(i)}{\mathit{\max}\left\{a(i),b(i)\right\}} $$

where *a*(*i*) is the average distance between the *i*th sample and all other samples included in the *j*th cluster, *b*(*i*) is the minimum average distance between the *i*th sample and all the samples clustered in kth cluster for *k* ≠ *j*. A point is regarded as well clustered if *s*(*i*) is large. The silhouette width is an internal cluster validity index used when true class labels are unknown. With a partitioning solution C, the silhouette width judges the quality and determines the proper number of partitions within a dataset. The overall average silhouette value can be an effective validity index for any partition. Choosing the optimal number of clusters/partitions is proposed as the value maximizing the average *s*(*i*) over the data set [19].

Connectivity was suggested in [14] as a clustering or partitioning validity measure such as

$$ Conn(C)={\sum}_{i=1}^n{\sum}_{j=1}^p{x}_{i,{nn}_i(j)} $$

where *C* = { *C*_1_, ⋯, *C*_*N*_} are clusters, and *p* is the number of variables contributing to the connectivity measure. Define *nn*_*i*_(*j*) is the *j*th nearest neighbor of observation *i*, and let ${x}_{i,{nn}_i(j)}\kern0.5em$be zero if *i* and *nn*_*i*_(*j*) are in the same cluster and *1/j* otherwise.

The connectivity assesses how well a given partitioning agrees with the concept of connectedness. This evaluates to what degree a partitioning observes local densities and groups genes (data items) together within their nearest neighbor in the data space based on violation counts of nearest neighbor relationships. The connectivity has a value between zero and ∞ that should be minimized for the best results. Dunn’s index [9] is another type of connectedness measure between clusters.

Stability measures can be computed after partitioning. Average Distance (AD) computes the average distance between genes placed in the same cluster by clustering based on the full data and clustering based on the data with a single column removed. AD has a value between zero and ∞; therefore, smaller values are preferred.

Figure of Merit (FOM) measures the average intra-cluster variance of the genes in the deleted column, where clustering is based on remaining (undeleted) samples. FOM estimates the mean error using predictions based on cluster averages. The final FOM score is averaged over all the removed columns with a value between zero and ∞. FOM with smaller values means better performance.

## Results and discussion

### Worked example

We consider flexible functional patterns of data since real gene expression functions are various with noise. Nonlinear curves are generated according to the regression model

$$ {Y}_{iu}={f}_i\left({t}_u\right)+\sigma {\varepsilon}_{iu} $$

for *i* = 1, 2, ⋯, 6,  *u* = 1, 2, ⋯, *m*, and *t*_*u*_ = *u*/*m*. The underlying regression functions for *f* are:

$$ {f}_1(t)=0 $$

$$ {f}_2(t)=\left(\frac{5-5t}{2}\right)\wedge \left(\ {\left(\frac{5t-2}{3}\right)}^2+\mathit{\sin}\left(\frac{5\pi t}{2}\right)\right) $$

$$ {f}_3(t)=20\left(t-0.1\right)\left(t-0.5\right)\left(t-0.7\right) $$

$$ {f}_4(t)=-2t+\mathit{\sin}\left(5\pi t/2\right) $$

$$ {f}_5(t)=2\mathit{\cos}\left(2\pi t\right) $$

$$ {f}_6(t)=2\left|t-0.3\right|. $$

The simulated data consist of 1000 curves with 6 different underlying functions. The data set has 500 curves of *f*_1_ and 100 curves of each of *f*_2_, ⋯, *f*_6_ to reflect certain aspect of gene expression data. Noise is imitated by adding random values from a normal distribution. Two noise levels are considered as low noise σ = 0.5 and high noise σ = 1.5. The number of time points is set to *m =* 20.

**Fig. 1:**
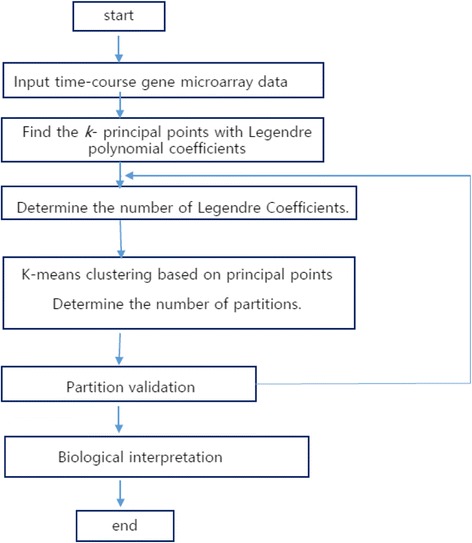
Flowchart of the whole methodology of the proposed partitioning

The advantages of the proposed method are evaluated by simulations. The number of subsets are known as K = 6. Table 1 shows connectivity and silhouette values after partitioning, which are better for 6 subsets with *J* = 3, 4, 5 coefficients in Gaussian-based principal points partitioning. The mean silhouette values and connectivity vary little according to *J* values. The number of subsets can be determined with modified GAP statistics [49]. The simulation results illustrate that the principal points via Legendre polynomial coefficients have favorable statistical properties in connectedness and can be used in time-course gene data. Figure 1 provides the flowchart of our proposed partitioning procedure.

**Table 1:** Comparison of partitioning with principal points for original data, Legendre polynomial coefficients and Fourier coefficients in 500 repetitions and *m =* 20 repeated design points with low noise σ = 0.5 and high noise σ = 1.5

	K = 6 subsets	σ = 0.5		σ = 1.5	
Number of coeff		Mean Silhouette	Connectivity	Mean Silhouette	Connectivity
*J* = 3	Original data: y	0.114	102.05	0.076	105.54
	Legendre coeff: LPC	0.531	25.036	0.511	23.932
	Fourier coeff: FC	0.270	61.628	0.235	63.621
*J* = 4	Original data: y	0.118	102.691	0.082	105.497
	Legendre coef: LPC	0.534	22.699	0.539	22.614
	Fourier coeff: FC	0.235	68.572	0.224	73.308
*J* = 5	Original data: y	0.116	101.743	0.081	105.343
	Legendre coeff: LPC	0.547	22.526	0.539	22.846
	Fourier coeff: FC	0.212	74.110	0.198	77.572

Evaluation for a clustering method can be done on theoretical grounds by internal or external validation, or both [14, 31]. Likewise, silhouette width and connectivity measure is considered for tightness in regards to genes in partitions. The evaluation of partitioning algorithms for gene data cannot be conducted by similar measures, but only by internal or external validation measures. The connectivity of genes in each partition can be regarded as an association of genes.

#### Application to partitioning with yeast cell cycle microarray expression data

The yeast cell-cycle data set [38] includes more than 6000 yeast genes at 18 time points measured every 7 min that start at 0 min and end at 119 min. Temporal gene expression data (α-factor synchronized) for the yeast cell cycle data is used for our real data analysis. A total of 4489 genes remain after removing genes with the missing values. The time-course yeast microarray data are functional data obtained according to 18 time points for each gene [38]. Yeast is a free living, eukaryotic and single cell and highly complex organism that plays an important role for biology research.

First, the Legendre coefficients and Fourier coefficients are estimated. Then each set of estimated coefficients is applied to K-means clustering and Gaussian-based principal point estimation with the estimated covariance matrix.

**Fig. 2:**
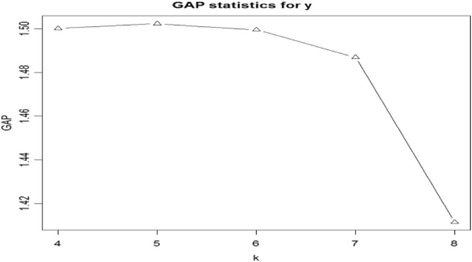
GAP statistics from K = 4 to K = 8

Figure 2 shows that the GAP statistic for original data is maximized at *k =* 5. We considered from *k =* 4 since previous research typically provides at least 4 subsets, even with different criterion. BIC is maximized at *k =* 5 for model-based clustering with the Legendre polynomial coefficients under VEV (volume:variable, shape:equal, and mean:variable) condition. Therefore, we decide the number of subsets as *k =* 5.

The number of Legendre polynomials *J* is considered from *J* = 2 to *J* = 7 and the average silhouette value is maximized at *J* = 5. The average silhouette values for *J* = 4 and *J* = 5 is 0.511 and 0.520 which are very close. However the mean within sum of squares (MSW) with *J* = 4 is 7376 and MSW with *J* = 5 is 144,650. MSW with *J* = 4 is less than MSW with *J* = 5. Consequently, the genes within each subset are closer to its center for *J* = 4. Therefore, we decide to use *J* = 4 Legendre polynomials and one constant term with the resulting coefficients used for partitioning. Table 2 shows that *J* = 4 Fourier coefficients are suggested for partitioning. We consider the same number of Fourier coefficients and those of Legendre polynomials for the comparison of yeast data.

**Table 2:** Principal points partitioning results in K = 5 subsets based on *J* the number Legendre polynomial coefficients and Fourier coefficients with yeast data

	LPC^a^	FC^b^
Number of LPC Number of FC	Average Silhouette	Average Silhouette
*J* = 2	0.485	0.2256
*J* = 3	0.494	0.1954
*J* = 4	0.511	0.2118
*J* = 5	0.520	0.1417
*J* = 6	0.516	0.1298
*J* = 7	0.500	0.1394

^a^LPC: Legendre polynomial coefficients

^b^FC: Fourier coefficients

**Fig. 3:**
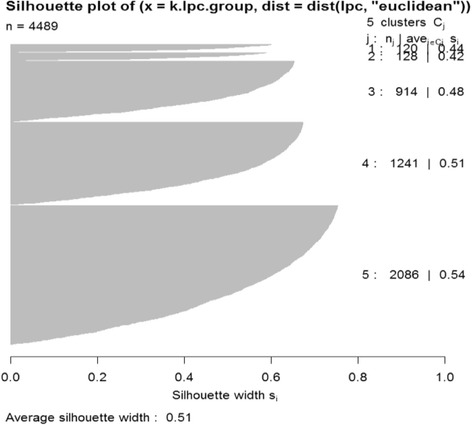
Silhouette values in 5 subsets with principal points partitioning with *J = 4* Legendre polynomial coefficients for yeast data

**Fig. 4:**
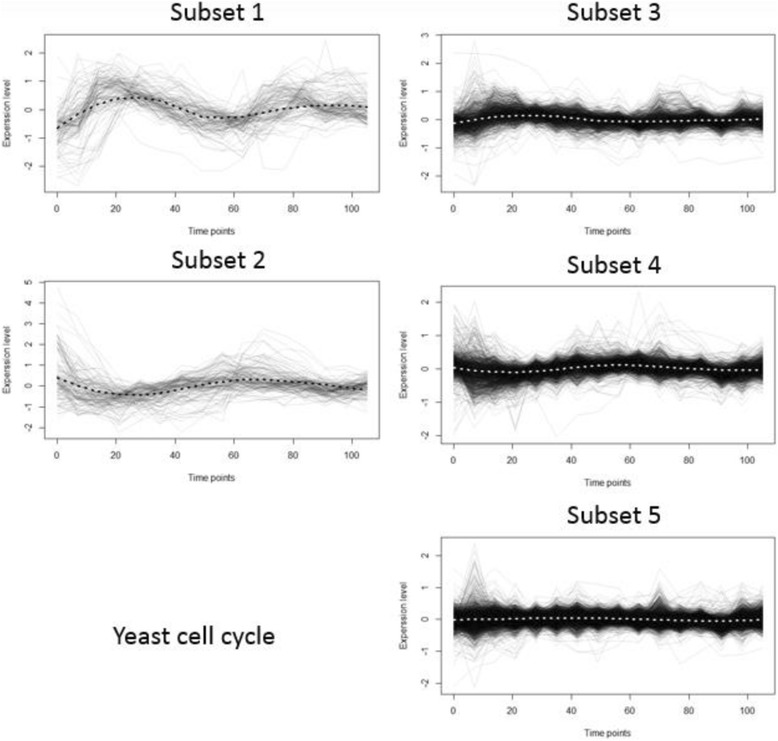
Loess smoothed gene score means in 5 subsets based on five Legendre polynomial coefficients of yeast data

**Fig. 5:**
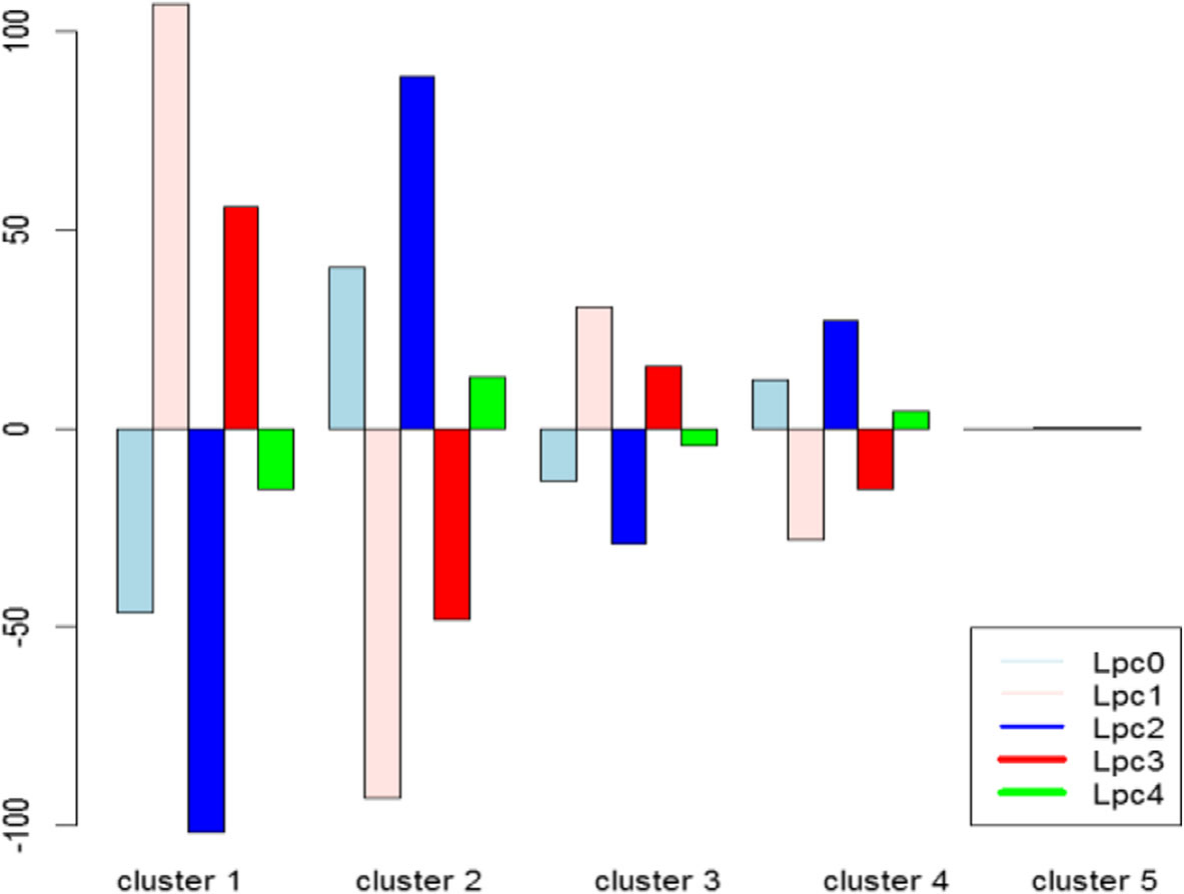
Means of Legendre polynomial coefficients in five subsets of yeast data

Then K-means clustering is done with the time-course original data (y), with 4 Legendre polynomial coefficients (LPC) and one constant term, and with 4 Fourier coefficients (FC) and one constant mean term respectively. K-means clustering with Legendre polynomials result in five subsets with 120, 128, 914, 1241, and 2086 genes respectively. The 2086 genes in Subset 5 seem to be nondifferential. Table 3 shows the partitioning results with the validation measures such as silhouette and connectivity. LPC has the best silhouette and the lowest (best) connectivity values. Figure 3 shows means, 2.5% and 97.5% percentiles of gene scores which provides a 95% empirical confidence interval for each subset. The graph in the bottom right-hand corner of Fig. 3 shows the estimated mean change patterns of the five subsets. Figure 4 and Fig. 5 provide the LPC partitioning information including underlying functions and Legendre polynomial coefficients. In Fig. 4, the expression patterns of Subset 1 and 2 are similar to those of Subset 3 and 4, respectively, with less fluctuations. This means their relevance to cell cycle could be similar to each other (Subset 1 and 3, Subset 2 and 4), but they are possibly involved in different biological activities during the cell cycle. Subset 3 and Subset 4 seem to have initial different parts and their coefficients are reverse in sign in Fig. 5. Our proposed algorithm was able to identify any subtle differences in terms of biological processes. In Table 4, most of the GO terms in Subset 1 are mainly related to DNA replication during the S (synthesis) phase of cell cycle, while the terms in Subset 3 represent different biological processes such as protein mannosylation, which is an essential process for cell wall maintenance. GO terms related to cell division, including cell wall synthesis, were in Subset 2, which is mainly activated during the M (mitosis) phase of the cell cycle. Genes in Subset 4 showed similar expression profiles with Subset 2, but their biological processes are mostly related to a protein synthesis that was not represented in Subset 2. Therefore, the genes in Subset 3 and 4 are possibly involved in the crucial biological processes required during the S or M phase of the cell cycle. The constant expression pattern and over-represented GO terms in the subsets suggested that these genes could be related to biological processes such as protein transport, which is constantly activated throughout the cell cycle.

**Table 3:** Principal points partitioning results with original data, Legendre polynomial coefficients and Fourier coefficients in K = 5 subsets with yeast data

K = 5	Components		
	Y (*m* = 18)	LPC (*J* = 4)	FC (*J* = 4)
Number of genes in 5 subsets	1232 743,484,147 1883	120,128,914 1241 2086	2625 495 40 1160 169
Average Silhouette	0.095	0.511	0.2118
Connectivity	2273.658	61.53	1018.696

**Table 4:** Summary of over-represented KEGG pathway terms in each subset of yeast data

Category (Annotated / Total, %)	Term	KEGG id	count	*p*-value	FDR (E-2: 10^−2^)
Subset 1 (36/106, 33%)	DNA replication	ko03030	10	6.10E-09	2.40E-07
	Mismatch repair	ko03430	7	2.20E-06	4.30E-05
	Cell cycle - yeast	ko04111	11	1.80E-04	2.30E-03
	Amino sugar and nucleotide sugar metabolism	ko00520	6	4.70E-04	4.70E-03
	Pyrimidine metabolism	ko00240	8	6.70E-04	5.40E-03
	Base excision repair	ko03410	4	6.00E-03	3.90E-02
	Nucleotide excision repair	ko03420	5	7.30E-03	4.10E-02
	Starch and sucrose metabolism	ko00500	5	9.60E-03	4.70E-02
	Galactose metabolism	ko00052	4	1.40E-02	5.90E-02
	Purine metabolism	ko00230	7	1.50E-02	5.90E-02
	Meiosis - yeast	ko04113	7	4.90E-02	1.70E-01
	Homologous recombination	ko03440	3	6.10E-02	1.90E-01
	Fructose and mannose metabolism	ko00051	3	7.30E-02	2.10E-01
Subset 2 (14/123, 11%)	MAPK signaling pathway - yeast	ko04011	6	6.00E-04	1.20E-02
	Cell cycle - yeast	ko04111	8	1.20E-03	1.20E-02
	Meiosis - yeast	ko04113	7	7.10E-03	4.90E-02
	DNA replication	ko03030	3	7.00E-02	3.20E-01
Subset 3 (195/821, 23%)	Metabolic pathways	map01100	136	3.90E-09	3.80E-07
	Biosynthesis of secondary metabolites	map01110	65	1.20E-05	5.80E-04
	Glycerophospholipid metabolism	ko00564	14	5.50E-04	1.70E-02
	Carbon metabolism	ko01200	29	5.70E-04	1.40E-02
	Tyrosine metabolism	ko00350	7	6.10E-03	1.10E-01
	Glycolysis / Gluconeogenesis	ko00010	16	6.70E-03	1.00E-01
	Propanoate metabolism	ko00640	6	9.30E-03	1.20E-01
	Fatty acid elongation	ko00062	5	1.40E-02	1.50E-01
	Biosynthesis of antibiotics	map01130	41	1.70E-02	1.70E-01
	Fatty acid metabolism	ko01212	8	1.90E-02	1.70E-01
	Oxidative phosphorylation	ko00190	17	2.30E-02	1.80E-01
	Pyruvate metabolism	ko00620	11	2.70E-02	2.00E-01
	Starch and sucrose metabolism	ko00500	11	3.20E-02	2.10E-01
	Glycosylphosphatidylinositol(GPI)-anchor biosynthesis	ko00563	8	3.90E-02	2.40E-01
	Mismatch repair	ko03430	7	4.00E-02	2.30E-01
	Phenylalanine metabolism	ko00360	5	4.70E-02	2.50E-01
	Biosynthesis of unsaturated fatty acids	ko01040	5	4.70E-02	2.50E-01
	Protein processing in endoplasmic reticulum	ko04141	18	5.50E-02	2.70E-01
	Arginine biosynthesis	ko00220	6	6.40E-02	3.00E-01
	MAPK signaling pathway - yeast	ko04011	12	6.60E-02	2.90E-01
	Methane metabolism	ko00680	8	6.70E-02	2.90E-01
	Degradation of aromatic compounds	ko01220	4	7.80E-02	3.10E-01
	Other types of O-glycan biosynthesis	ko00514	5	8.20E-02	3.10E-01
	N-Glycan biosynthesis	ko00510	8	9.20E-02	3.30E-01
	Fatty acid degradation	ko00071	6	9.60E-02	3.30E-01
Subset 4 (191/1113, 17%)	Ribosome biogenesis in eukaryotes	ko03008	33	2.40E-05	2.30E-03
	RNA transport	ko03013	34	2.40E-05	1.20E-03
	Purine metabolism	ko00230	34	5.10E-05	1.70E-03
	RNA polymerase	ko03020	15	2.40E-04	5.70E-03
	Steroid biosynthesis	ko00100	9	5.20E-03	9.50E-02
	Biosynthesis of amino acids	ko01230	33	1.30E-02	1.80E-01
	Proteasome	ko03050	13	1.40E-02	1.80E-01
	Non-homologous end-joining	ko03450	6	2.00E-02	2.20E-01
	Pyrimidine metabolism	ko00240	21	2.20E-02	2.20E-01
	RNA degradation	ko03018	18	3.30E-02	2.80E-01
	Cysteine and methionine metabolism	ko00270	12	4.30E-02	3.20E-01
	Phosphatidylinositol signaling system	ko04070	7	5.00E-02	3.40E-01
	Biosynthesis of antibiotics	map01130	49	6.00E-02	3.70E-01
Subset 5 (407/1809, 22%)	Metabolic pathways	map01100	239	2.60E-05	2.70E-03
	Biosynthesis of secondary metabolites	map01110	113	1.90E-04	1.00E-02
	Protein processing in endoplasmic reticulum	ko04141	40	6.50E-04	2.20E-02
	Biosynthesis of antibiotics	map01130	84	1.40E-03	3.60E-02
	Basal transcription factors	ko03022	18	3.10E-03	6.30E-02
	mRNA surveillance pathway	ko03015	23	4.50E-03	7.50E-02
	Endocytosis	ko04144	31	9.50E-03	1.30E-01
	Ubiquitin mediated proteolysis	ko04120	22	1.40E-02	1.70E-01
	Spliceosome	ko03040	33	1.50E-02	1.70E-01
	Phagosome	ko04145	17	3.20E-02	2.90E-01
	Biosynthesis of amino acids	ko01230	46	3.40E-02	2.80E-01
	Glycine, serine and threonine metabolism	ko00260	15	5.00E-02	3.60E-01
	Citrate cycle (TCA cycle)	ko00020	15	5.00E-02	3.60E-01
	Arginine and proline metabolism	ko00330	11	5.20E-02	3.50E-01
	Proteasome	ko03050	16	5.20E-02	3.30E-01
	Phenylalanine, tyrosine and tryptophan biosynthesis	ko00400	9	8.50E-02	4.60E-01
	Glyoxylate and dicarboxylate metabolism	ko00630	12	9.80E-02	4.90E-01
	Valine, leucine and isoleucine biosynthesis	ko00290	7	9.90E-02	4.80E-01

**Fig. 6:**
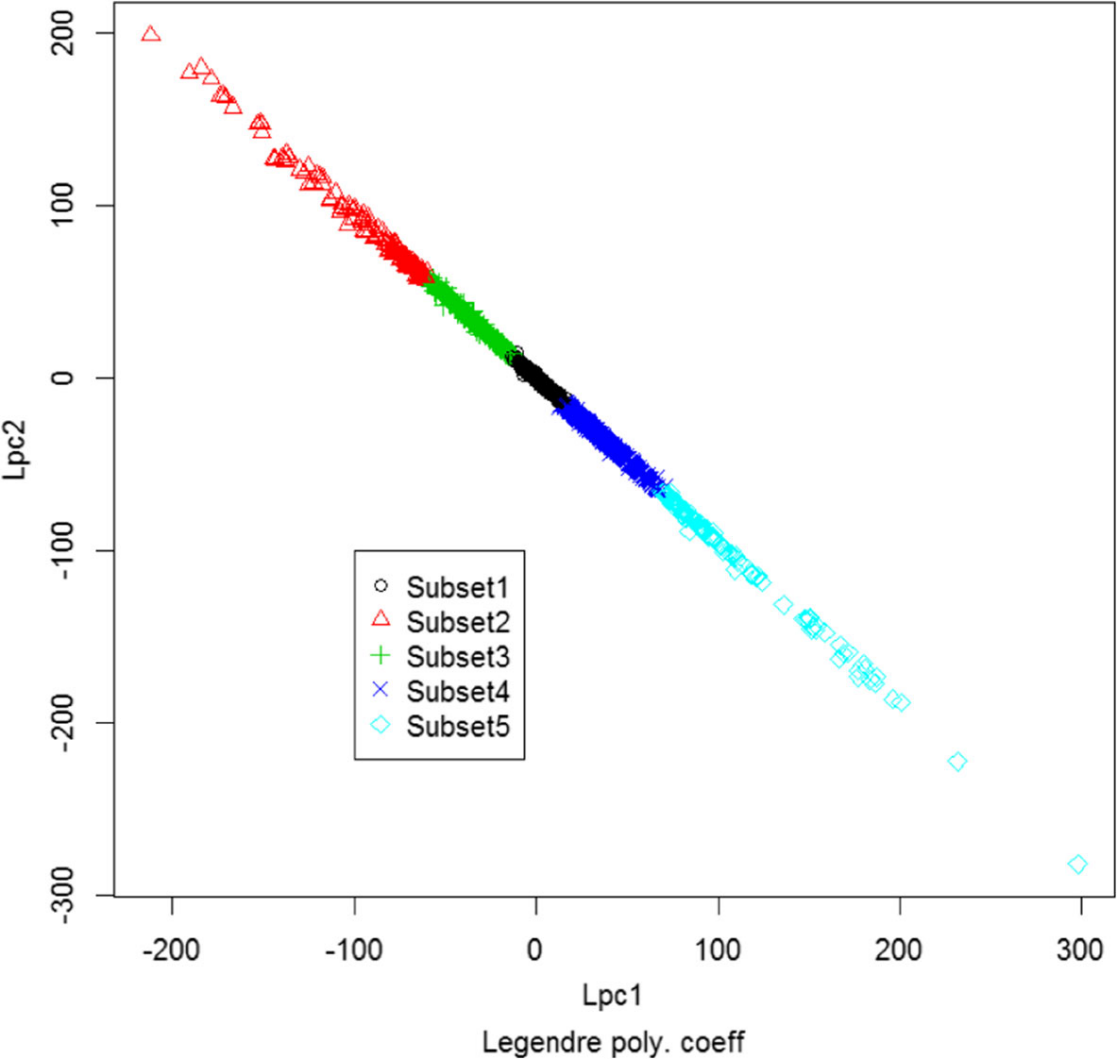
Plot of linear and quadratic coefficients $\left({\widehat{\beta}}_{i1},{\widehat{\beta}}_{i2}\right)$ for Legendre polynomials in each subset of yeast data

**Fig. 7:**
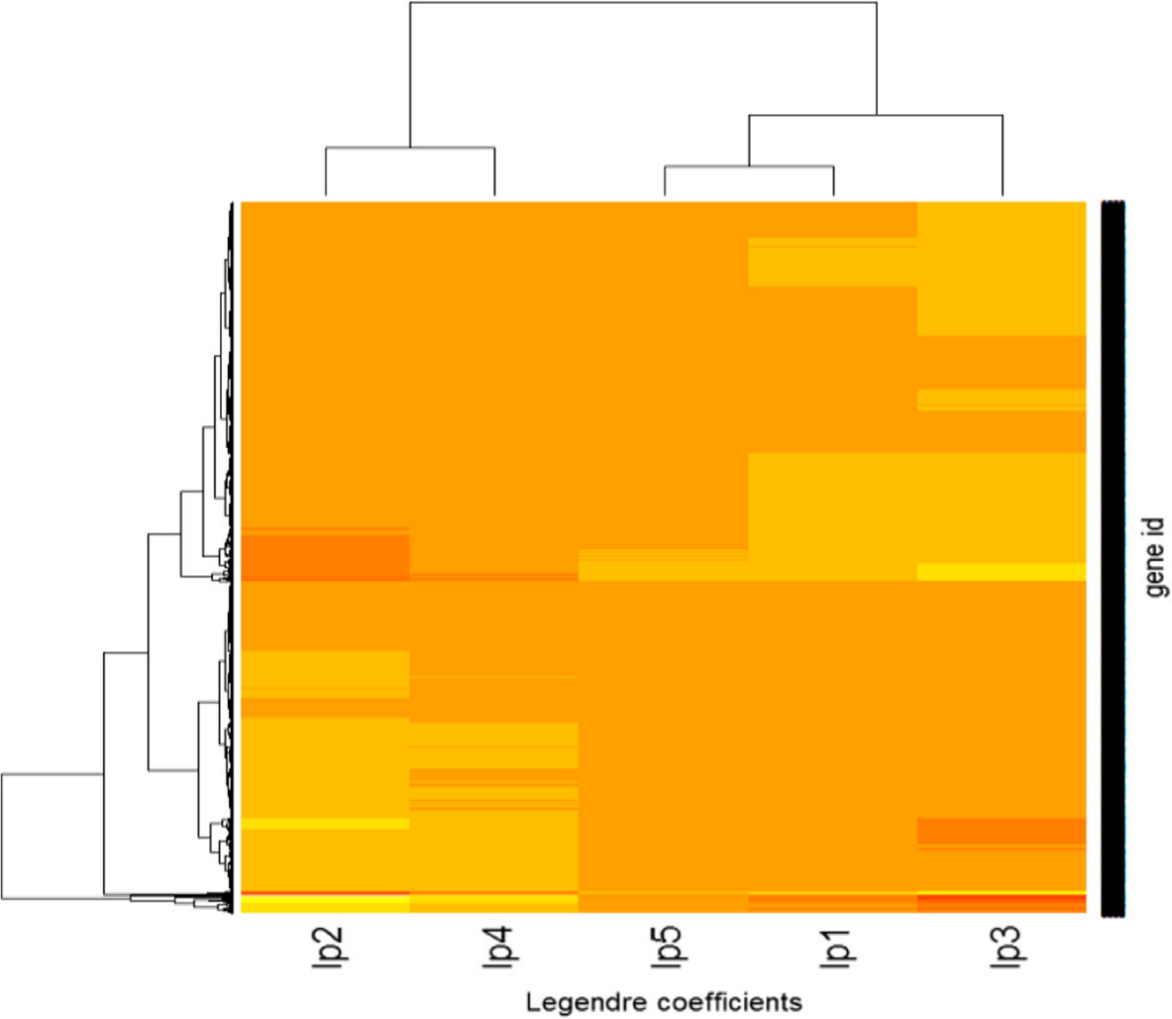
Heatmap of Legendre polynomial coefficients of yeast data

Nonparametric estimators of principal points are given by the subset center means (Fig. 5). Figure 6 shows the relation between linear and quadratic Legendre polynomial coefficients. Figure 7 shows the hierarchical structure of Legendre coefficients as the heatmap. Legendre coefficients 2 and 4 as well as coefficients 1 and 3 seem to be clustered first. Subset stability measures such as average distance (AD) and Figure of Merit (FOM) are computed. AD is 20.6059 and FOM is 8.15, which are minimized with 5 subsets instead of 4 subsets; consequently, 5 partitions are more stable than 4 partitions in regards to AD and FOM.

**Fig. 8:**
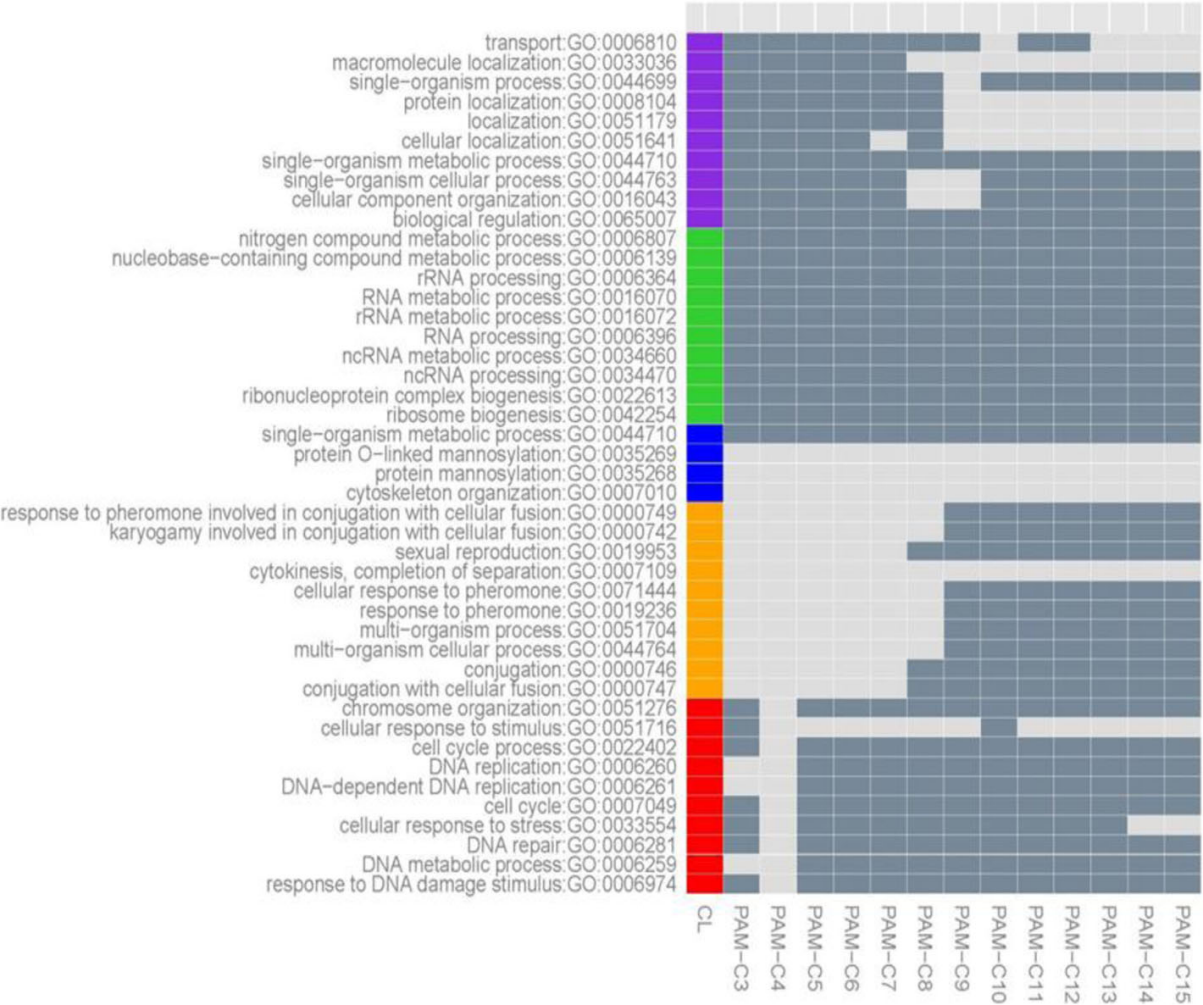
Top 10 over-represented GO terms in each subset (Subset 1:Red, Subset 2:Orange, Subset 3:Blue, Subset 4:Green, and Subset 5:Purple) in yeast data. Only Subset 3 has four over-represented GO terms. For the comparison, PAM was performed with various numbers of centers ranging from 3 to 15. The cell is colored with dark gray or light gray if PAM found the same GO terms with ORA test

Over-Represented Analysis (ORA) was performed with the genes in each subset in order to explain the explain biological relevance of the partitioned data. ORA searches for Gene Ontology (GO) terms of a given set of genes by evaluating the statistical significance of over-represented functional and molecular mechanisms [5, 6]. GO is divided into three separate ontologies (Cellular Component, Molecular Function, and Biological Process) and our ORA analysis focuses on the Biological Process of a group of genes. In each subset, we selected the top 10 over-represented GO terms in the smallest order of *p*-values and compared them in terms of biological significance to over-represented GO terms with the Partitioning Around Medoids (PAM) clustering method (Fig. 8) that can be seen in detail in the legend of the figure. Many of the annotated GO terms, such as DNA replication in Subset 1 and conjugation in Subset 2, are adequate to explain the cell cycle data used. However, in Subset 3, our partitioning technique found four GO terms, GO:0007010, GO:0035268, GO:0035269, and GO:0044710 were not significantly over-represented in the PAM result. In Subset 3, the annotations of the found terms, especially Protein O-linked mannosylation recently reported that the lack of this biological function crucially affects cell morphology such as cell wall defects and cell-cell separation in S. pombe [50]. Therefore GO: 0035268, GO: 0035269, and GO:0044710 are closely related to each other and reasonably explain the cell cycle process. In addition, GO:0035268 and GO:0035269 can be found as child terms by following connections from GO:0044710 in a GO tree. The results indicate that our partitioning approach can find functionally related genes which are not identified by the commonly used PAM clustering method.

With similar approach, we annotated the genes in each subset in terms of biological pathways. KEGG is a well-known pathway whose biological functions are manually curated [18]. DAVID website provides KEGG information along with various annotation tools that include ORA [16]. Table 4 summarizes the over-represented KEGG pathways that are statistically significant with *p*-value <0.1. We drew our attention on Subset1 where the highly significant pathway terms are involved in DNA replication and repair processes during the cell cycle. Sugar metabolisms are easily detected because sugars are the basic building blocks of DNA. From these annotation results, the genes in Subset 1 are closely interrelated in the role of DNA replication. However, 53 of 96 genes in this subset are not included for the annotation; therefore, these 53 genes could be good candidates for further study with a hypothesis that they are dynamically involved in the DNA replication and repair process. Recently FDA ([33, 35]) provides new tools well-suited for discrimination and classification [30, 42].

#### Application to partitioning with *Escherichia coli* microarray expression data

We applied our method to microarray data tracking *Escherichia coli (E. coli)* transcriptional responses to recovering from the stationary phase. This experimental dataset consists of log ratio intensity values for *E. coli* genes measured in cDNA microarray hybridizations. The final data set includes more than 3607 genes at 11 time points; however, 3452 genes remain after removing genes with missing values. Time-course *E. coli* microarray data are regarded as functional data obtained according to 11 time points for each gene. This dataset is part of a study that tracks transcriptional responses to over 30 chemical and physiological perturbations [34].

**Fig. 9:**
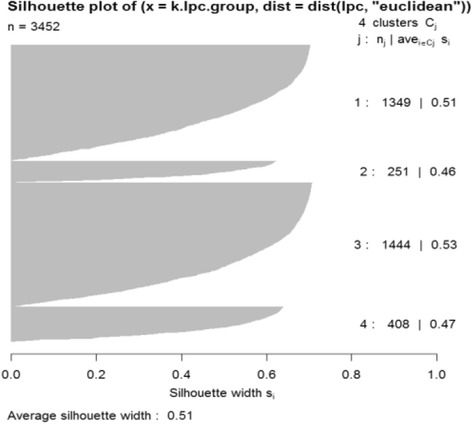
Silhouette values in 4 subsets with principal points partitioning with *J* = 4 Legendre polynomial coefficients of *E.coli* data

**Fig. 10:**
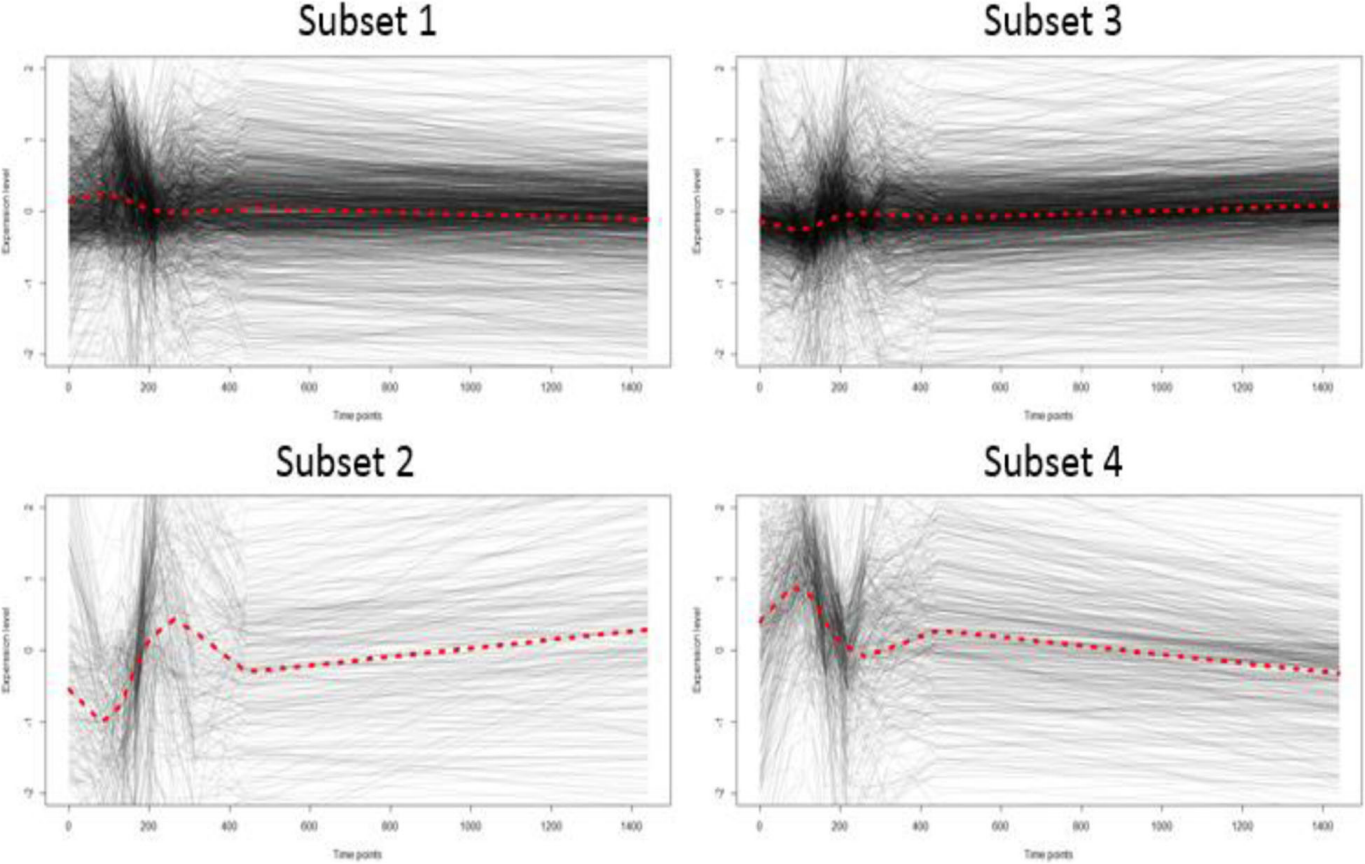
Expression patterns of genes in each subset. Red lines represent smoothed gene expression mean of *E.coli* microarray data

The current study took advantage of the available information about the physiology of *E. coli* bacteria. Functional and regulatory classifications for *E. coli* genes are considered to evaluate transcriptional activity within and across groups of related genes. Figure 9 provides the silhouette profiles of the partitioning with the overall average silhouette value at 0.51. Figure 10 shows the expression patterns of the four subsets that were determined by the proposed algorithm. Each subset has 1349, 251, 1444, and 408 genes from Subset 1 to Subset 4. The connectivity measure is 62.68 and Dunn’s index is 0.000759 for the resulting partitions. Each subset has its own distinctive expression pattern depicted by the smoothed expression mean (red line). Similar to the yeast cell cycle data results, Subset 1 and Subset 3 have identical expression profiles to Subset 4 and Subset 2, respectively, but with less fluctuations. The genes in Subset 4 and Subset 1 are actively involved in recovery processes such as protein synthesis, carbon energy metabolism, cell division, and nutrient uptake; however, the genes in Subset 2 and Subset 3 were possibly involved in the processes that stabilize the cells after their growth [34]. We performed gene enrichment analysis using the DAVID website to evaluate the partitioned genes in terms of their encoded protein keywords. Table 5 shows the enriched keywords with *p*-values less than 0.01. As expected, genes in Subset 4 are mainly involved in cell growth; however, the genes in Subset 1 are also related to cell growth similar to genes in Subset 4 that have distinct cellular processes such as molecular bindings. However, the keywords in Subset 2 and Subset 3 are mainly related to enzymatic processes after cell growth. For example, acetylation affects protein stability; in addition, purine/pyrimidine biosynthesis, ligase, transferase, are all important enzymatic processes for cell stabilization. Oxidoreductase and NADP are also responsible for the electron transfer. The proposed technique proved that it provides decisive and biologically meaningful subsets of genes in time-course experiments despite the limited biological annotations.

**Table 5:** Summary of over-represented GO terms (molecular function) in each subset of *E.coli* data

Subset4 (339/402, 84%)	Transposition	26	3.10E-12
Category (Annotated / Total, %)	Term	Count	*p*-value
Subset1 (932/1320, 70%)	DNA recombination	49	8.80E-06
	RNA-binding	58	2.00E-04
	Transposition	31	2.60E-04
	Protein biosynthesis	26	3.60E-04
	Transposable element	31	3.90E-04
	tRNA-binding	16	7.90E-04
	DNA-binding	159	1.70E-03
	tRNA processing	26	2.90E-03
	Nucleotide-binding	180	2.90E-03
	ATP-binding	155	2.90E-03
	Cytoplasm	232	4.30E-03
	Nucleotidyltransferase	23	6.90E-03
Subset2 (188/248, 75%)	Acetylation	21	6.20E-07
	Purine biosynthesis	9	2.10E-06
	Oxidoreductase	43	6.10E-06
	Nitrate assimilation	8	6.50E-05
	Metal-binding	59	2.40E-04
	Ligase	15	9.20E-04
	Tricarboxylic acid cycle	7	1.40E-03
	Pyridoxal phosphate	11	2.20E-03
	NADP	14	2.30E-03
	Pyrimidine biosynthesis	5	2.90E-03
	Enterobactin biosynthesis	4	6.10E-03
	Transferase	48	8.80E-03
Subset3 (609/1377, 44%)	Oxidoreductase	137	5.80E-03
	Iron-sulfur	58	7.40E-03
Subset4 (339/402, 84%)	Transposition	26	3.10E-12
	Transposable element	26	5.20E-12
	DNA recombination	29	6.30E-09
	Cytoplasm	96	2.80E-06
	Ribonucleoprotein	16	2.00E-04
	Bacterial flagellum	9	2.90E-04
	Ribosomal protein	15	5.70E-04
	Transmembrane beta strand	13	6.00E-04
	RNA-binding	24	9.80E-04
	DNA replication	12	1.20E-03
	Cell outer membrane	18	2.30E-03
	Ion transport	21	3.20E-03
	Bacterial flagellum biogenesis	7	3.60E-03
	rRNA processing	10	4.50E-03
	Methyltransferase	13	7.40E-03

## Conclusions

The dynamic nature of biological systems makes the investigation of temporal gene expression data important for exploration of gene expression regulation since they provide valuable functional information about temporal underlying patterns. Partitioning these genes is therefore an interesting problem in order to find gene functions in each partition.

In this paper, we present a functional partitioning procedure using principal points for temporal gene expression data after Legendre polynomial transformation. The optimal partitioning results produce a set of gene curve profiles that identify distinct types of gene expressions. Temporal gene expression data can be viewed as functional data since they are continuous and discretized samples of smooth random gene expression trajectories according to time. Partitioning differentiates cell-cycle regulated genes and other non-cell-cycle regulated genes for yeast. Also partitioning differentiates distinct cellular processes for *E. coli*.

The proposed method identified each partition for its cellular process properties, which shows that transformation via orthogonal polynomials could work for self-consistent partitioning. Our contributions include proposing principal points for microarray partitioning and the idea of some functional coefficients as transformation giving information about functional data. The future development of our method considers other transformations of functional data and functional time dependency that expects improvements in partitioning evaluation.

The yeast cell cycle data used is an early version of a two channel microarray that was hybridized with cDNA from two samples to be compared (e.g. normal versus cancer cells). The *E. coli* dataset in this work is also generated using the custom made two channel microarray technique with two different fluorescence dyes. However, RNA-Seq uses a next-generation sequencing (NGS) technique to measure the quantity of RNA in a sample of interest. The expression intensity is quantified by counting the number of reads mapped to each gene; therefore, care should be taken as the changes of total RNA amount between conditions possibly lead misrepresentation of the changes of individual transcript. In conclusion our method can be applied if the RNA-Seq data is appropriately processed. Further study is expected to utilize the proposed method in the analysis of more complex model organisms such as rats.

## Abbreviations

AD: average distance
*E. coli*: *Escherichia coli*
FDA: functional data analysis
FOM: Figure of Merit
GO: Gene Ontology
GPR: Gaussian process regression
NGS: next-generation sequencing
ORA: Over-Represented Analysis
PAM: Partitioning Around Medoids
